# Response of Osteoblasts on Amine-Based Nanocoatings Correlates with the Amino Group Density

**DOI:** 10.3390/molecules28186505

**Published:** 2023-09-07

**Authors:** Susanne Seemann, Manuela Dubs, Dirk Koczan, Hernando S. Salapare, Arnaud Ponche, Laurent Pieuchot, Tatiana Petithory, Annika Wartenberg, Susanne Staehlke, Matthias Schnabelrauch, Karine Anselme, J. Barbara Nebe

**Affiliations:** 1 Institute for Cell Biology, Rostock University Medical Center, 18057 Rostock, Germanybarbara.nebe@med.uni-rostock.de (J.B.N.); 2 Department of Biomaterials, INNOVENT e.V., 07745 Jena, Germany; md1@innovent-jena.de (M.D.); aw@innovent-jena.de (A.W.); ms@innovent-jena.de (M.S.); 3 Department of Immunology, Rostock University Medical Center, 18057 Rostock, Germany; dirk.koczan@med.uni-rostock.de; 4 Institut de Science des Matériaux de Mulhouse (IS2M), CNRS, Université de Haute-Alsace, UMR 7361, 68100 Mulhouse, Francearnaud.ponche@uha.fr (A.P.); laurent.pieuchot@uha.fr (L.P.); tatiana.petithory@uha.fr (T.P.); karine.anselme@uha.fr (K.A.); 5 Department Life, Light & Matter, Interdisciplinary Faculty, University of Rostock, 18059 Rostock, Germany

**Keywords:** surface modification, amino coating, polymer, material characterization, surface charge, wettability, spreading, actin cytoskeleton, microarray, gene expression

## Abstract

Increased life expectancy in industrialized countries is causing an increased incidence of osteoporosis and the need for bioactive bone implants. The integration of implants can be improved physically, but mainly by chemical modifications of the material surface. It was recognized that amino-group-containing coatings improved cell attachment and intracellular signaling. The aim of this study was to determine the role of the amino group density in this positive cell behavior by developing controlled amino-rich nanolayers. This work used covalent grafting of polymer-based nanocoatings with different amino group densities. Titanium coated with the positively-charged trimethoxysilylpropyl modified poly(ethyleneimine) (Ti-TMS-PEI), which mostly improved cell area after 30 min, possessed the highest amino group density with an N/C of 32%. Interestingly, changes in adhesion-related genes on Ti-TMS-PEI could be seen after 4 h. The mRNA microarray data showed a premature transition of the MG-63 cells into the beginning differentiation phase after 24 h indicating Ti-TMS-PEI as a supportive factor for osseointegration. This amino-rich nanolayer also induced higher bovine serum albumin protein adsorption and caused the cells to migrate slower on the surface after a more extended period of cell settlement as an indication of a better surface anchorage. In conclusion, the cell spreading on amine-based nanocoatings correlated well with the amino group density (N/C).

## 1. Introduction

Demographic change and the associated increase in life expectancy in industrialized countries have increased osteoporosis incidence. This situation leads to more orthopedic operations and an increased need for bone and joint implants, representing a major financial burden for the health system [1,2]. Regarding good biocompatibility, mechanical properties, and corrosion resistance, implants made of metal such as titanium (Ti) or titanium alloys are preferred. A disadvantage of metallic implants is low osseointegration, which reduces the long-term stability of the implants [3]. In the frequently older recipients of orthopedic implants, the regenerative capacity of the bone tissue is often limited, which also impairs the osseointegration, and may lead to aseptic implant loosening [1,4,5].

The integration of implants can be improved by physical but mainly by chemical modifications of the implant surface. For example, titanium coated with triple helical peptides from type I collagen induced higher ALP activity and calcium content in bone marrow stromal cells. In rat tibia, these implants showed increased bone-implant contact [6,7]. The initial step in osseointegration is the adherence of osteoblasts [8]. The physico-chemical stimuli induced by the chemical surface of the biomaterial can affect cell adhesion and spreading [9,10]. It is known that amine-based coatings improve initial cell attachment, cell spreading, cell migration, and intracellular signaling [9,11] and allow the cells to melt into geometrically and stochastically created convex surface structures [2,12]. Solely these thin films rich in amine chemical groups (−NH_2_) promoted osteogenic differentiation in adipose-derived stem cells versus carboxyl (−COOH), methyl (−CH_3_), and hydroxyl (−OH) chemical groups [13]. The mechanism behind these impressive effects is the electrostatic interaction of positively charged amino groups and the cells’ net negative charge due to the hyaluronan coat and other membrane components of the cells [14,15,16]. Plasma-polymerized amine-based coatings improve the osseointegration of titanium implants in vivo by increasing bone-to-implant contact [17]. Already in the first 5 min after cell contact, more than twice as many cells can adhere to the surface of an amine-based surface coating than to coatings with extracellular matrix proteins [18]. These striking effects make amine-based coatings on titanium surfaces so interesting for implant research. However, there is limited information on the distinct cellular responses induced by specific amino group contents at the biomaterial interface.

Another hypothesis for the cause of the positive effects of the amino-rich organic layers is the increased adsorption of serum proteins such as vitronectin or fibronectin and the resulting enhanced interaction of cellular integrin receptors with the coated surface [19,20]. Several studies reported that amino-functionalized surfaces also have a high adsorption capacity of bovine serum albumin (BSA) due to the protein’s high stability and varying surface charges under neutral or acidic conditions [21,22,23]. These proteins adsorbed on the surface are essential in the subsequent interaction of an implant with cells, and the protein density on the surface dictates how cells would behave, such as how they would spread or migrate on the surface [24,25].

Another effect of amine-based surfaces is on cell migration. The chemically-influenced cell migration, called chemotaxis, is essential to investigate since biochemistry is mainly known to affect cells in their migratory behavior [26,27,28]. To observe this effect, live cell imaging coupled with different chemical characterizations is usually employed to correlate other cell characteristics and behaviors, such as changes in the cell speed, cell trajectory, and cell shape [29,30].

The amine-based nanocoatings used in this work were specifically chosen to cover and investigate a wide range of different amino group densities. In addition, different topographical properties were selected to study the influence of the surface-structural differences of the coatings on the physico-chemical properties and biological effects. As already mentioned in recent reports [2,9,11,12], smooth and thin films of positively charged polymerized allylamine (PPAAm) films with relatively high N/C ratios of about 20–26% deposited on various biomaterial implants as titanium or even ceramic surfaces showed optimal conditions for adhesion and spreading of human osteoblasts. Based on these positive effects, we studied different amino-group-containing nanolayers. In a first attempt, we prepared surface coatings bearing aminosilanes like (3-aminopropyl)triethoxysilane (APTES) [31] and N-(2-aminoethyl)-3-aminopropyltrimethoxysilane (2AE-APS). While APTES has primary amino groups in the molecule, 2AE-APS has both primary and secondary amino groups. In further studies, we used different polyethyleneimine (PEI)-based silane coatings [14]. PEIs are well-known for their highly positive charge and diverse structures, constituting a category of the most extensively developed polycations [32]. PEIs are found in different types: linear PEI (lPEI), which contains only primary and secondary amino groups, and branched PEI (bPEI), including mixtures of various primary, secondary, and tertiary amino groups. In this work, coatings with lPEI and bPEI were synthesized starting from (3-glycidyloxypropyl)trimethoxysilane (GOPTS)-bearing layers by treatment of the epoxide groups with lPEI and bPEI, respectively. In addition, a commercially available trimethoxysilylpropyl-modified polyethyleneimine (TMS-PEI) [33] was used.

The processes initiated in osteoblasts after contact with the implant surface are orchestrated by finely tuned-transcriptional processes. In the initial phase of osseointegration, cell adhesion and spreading are regulated [8]. Subsequently, transcriptional changes lead to the initiation of proliferation and, in the next phase, to the downregulation of proliferation and the initiation of differentiation of osteoblasts to osteocytes [34]. The surface properties of implant materials induce specific cellular signaling cascades and transcriptional changes. In order to improve early osseointegration, not only the enhancement of physical cell attachment should be addressed, but also possibilities to modulate the corresponding downstream signaling pathways. Therefore, it is important to understand the molecular and transcriptional mechanisms underlying the effects of chemical surface modifications of bone implants. So far, there is little information on which transcriptional changes the improved osseointegration by amine-based surfaces is based on.

This work focused on the influence of different amino group densities of nanocoatings (i) on the physico-chemical surface properties such as water contact angle, zeta potential, and element composition via XPS, as well as (ii) on the cell biological effects. The parameter cell spreading and fold change of Col I were the criteria for further selective experiments, i.a., gene expression, migration speed, traveled distance, and protein adsorption. The general aim was to identify the optimal amino group density for the adherence and gene expression of osteoblasts. In this innovative study, first, systematically prepared amine-based nanocoatings were chemically characterized and correlated with the cell-spreading capacity; and in a second step, the nanolayer with the highest correlation of the N/C value with cell spreading was analyzed in more detail on molecular and transcriptional processes. Understanding the mechanism of action of these amino-based nanolayers should provide fundamental knowledge for improving bioactive implants for systemically diseased bone.

## 2. Results

### 2.1. Chemical Characterization of Amine-Based Polymer Coatings

Physico-chemical results of the different amine-based nanocoatings concerning wettability and surface charge are given in Table 1.

Wettability: The analysis indicated a decrease in WCA values of the amine-based nanocoatings except Ti-2AE-APS. The Ti-Ref and Ti-2AE-APS showed slightly hydrophobic characteristics with a WCA of θ~95°. The other nanocoatings were hydrophilic, ranking as follows: Ti-APTES-100 > Ti-APTES-1 > Ti-GOPTS-bPEI > Ti-TMS-PEI > Ti-Col I > Ti-GOPTS-lPEI, with WCA values of θ 77°, 69°, 55°, 54°, 53°, and 51°, respectively.

Zeta potential: The ζ-potential could be changed due to amine-based nanocoatings compared with Ti-Ref at –81 mV. Negative surface potentials were found for Ti-GOPTS-lPEI < Ti-GOPTS-bPEI < Ti-APTES-1 < Ti-2AE-APS < Ti-Col I, with −49, −34, −21, −11, and −3 mV, respectively. Only two coatings present a positive ζ-potential and can be classified as moderately (Ti-TMS-PEI: +12 mV) and highly positive (Ti-APTES-100: +51 mV).

Therefore, the coating Ti-TMS-PEI displayed optimal surface properties for cell adherence and growth, because we found both a moderately positive surface charge of ζ +11.7 mV and a water contact angle of θ 53.6° [14].

### 2.2. N-Dependent Cell Spreading

**Figure 1:**
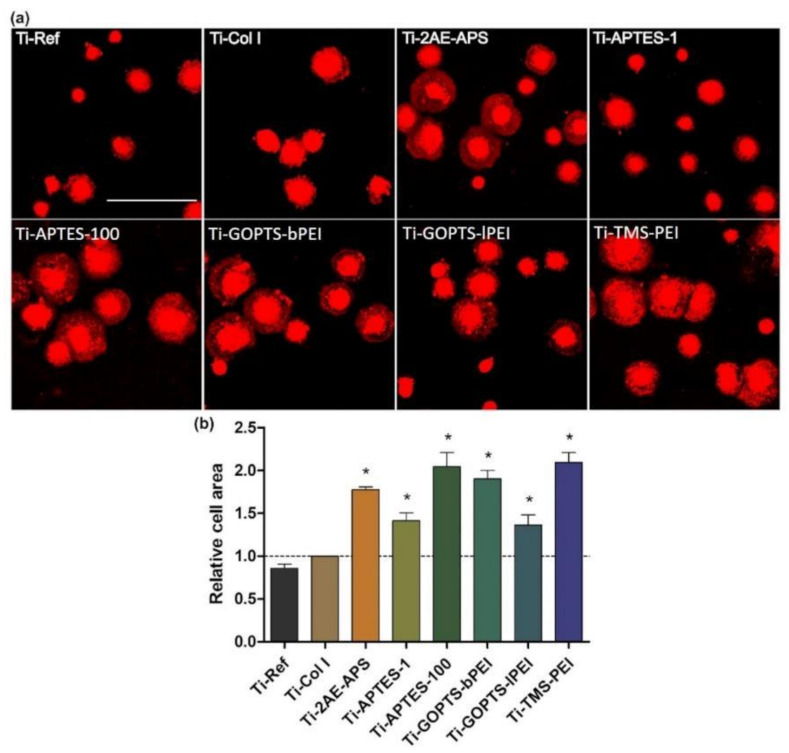
Spreading capacity of MG-63s on amine-based coatings after 30 min. (**a**) Cell area of membrane-stained cells (red). Note that cell areas increased on all amino-rich nanolayers compared to pure Ti-Ref and the extracellular matrix protein Col I as controls. (confocal microscopy, LSM 780, Carl Zeiss; red: PKH-26; scale bar 100 μm; abbreviations see Table 1). (**b**) Relative cell areas—the spreading values were compared to Col I (mean ± s.e.m.; n = 3 independent experiments with 50 cells each; unpaired *t*-test; * *p* < 0.05).

In the next step, we screened the different coatings for a substantial impact on cell adherence and growth. To verify the short-term effects on cell spreading, we analyzed the cell areas of MG-63s after 30 min seeding. The cell area of MG-63s enlarged on all amino-group-containing coatings compared to the collagen control (Figure 1, Table S1). Only the GOPTS-lPEI could not induce a significantly increased cell area of MG-63s. TMS-PEI showed the strongest enlargement of the cell area.

To compare the amino group density of the different coatings, we measured the element composition via XPS and calculated the N/C ratio. This analysis revealed a chemical structure-dependent correlation of the amino group density and the cell area of MG-63s (Table 2 and Table S2). TMS-PEI, which mostly improved cell area, possessed the highest amino group density. Therefore, for all further cell physiological experiments, we focused on this Ti coated with trimethoxysilylpropyl modified poly(ethyleneimine) (Ti-TMS-PEI).

### 2.3. Cell Morphology on Ti-TMS-PEI

**Figure 2:**
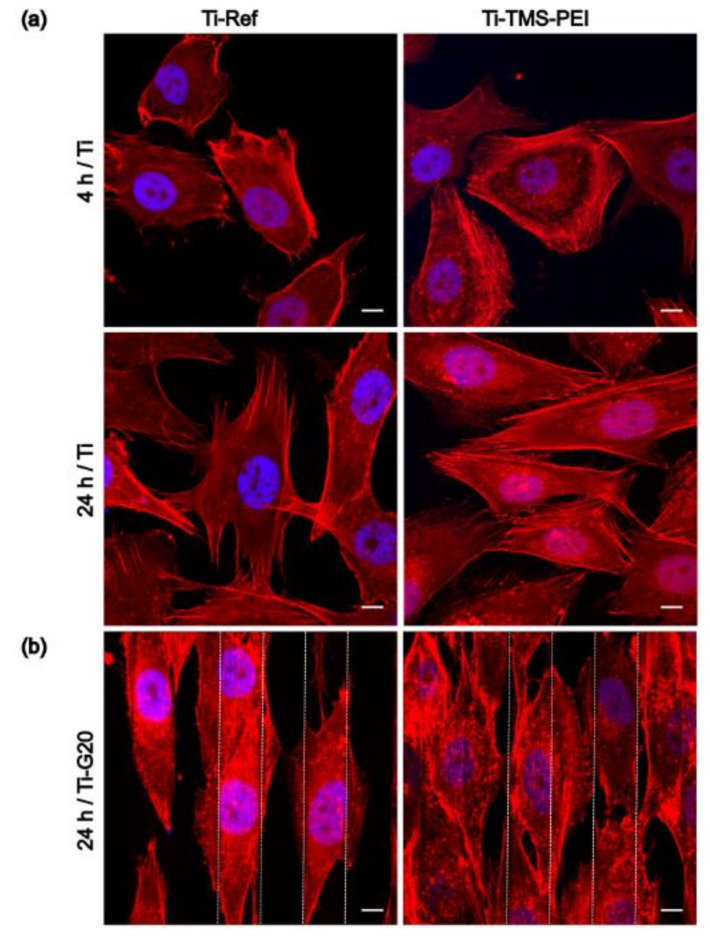
Actin cytoskeleton of MG-63 cells on amine-based Ti-TMS-PEI vs. uncoated Ti. (**a**) Cell spreading and development of actin filaments in the time frame 4 h and 24 h on planar Ti samples. MG-63s showed an increased cell area on Ti-TMS-PEI after 4 h compared to Ti-Ref, and the actin filaments are more pronounced. After 24 h, these differences in cell size and filament organization are equalized (representative figures of n = 3 independent experiments). (**b**) Cell orientation and actin formation of MG-63s on micro-grooved Ti-G20 structures after 24 h. Cells on grooved Ti-Ref samples (20 µm in width, 2 µm in height) appear aligned due to contact guidance. In contrast, cells on grooved Ti-TMS-PEI experience a slight abrogation of the contact guidance, i.e., the nanolayer has an impact that is dominant over the micro-structure (3D z-stack, dashed lines indicating groove edges, representative figures of n = 2 independent experiments). (For all images: confocal microscopy, LSM 780, Carl Zeiss; actin: phalloidin-TRITC, red, nucleus: DAPI, blue; scale bars 10 μm).

In the following, the cell biological effects of the most effective surface coating should be investigated in more detail. For this purpose, cell morphology on Ti-TMS-PEI was examined by confocal microscopy using actin staining. In order to establish comparability with the microarray analyses that were also performed, the cultivation period was set at 4 h and 24 h, respectively. Even after 4 h, a larger cell area was still evident on the Ti-TMS-PEI samples, whereas the difference had relativized after 24 h (Figure 2). The amino groups of Ti-TMS-PEI let the cells overcome surface guidance of a grooved Ti surface with 20 µm × 2 µm (width × height) dimension (Figure 2b).

**Figure 3:**
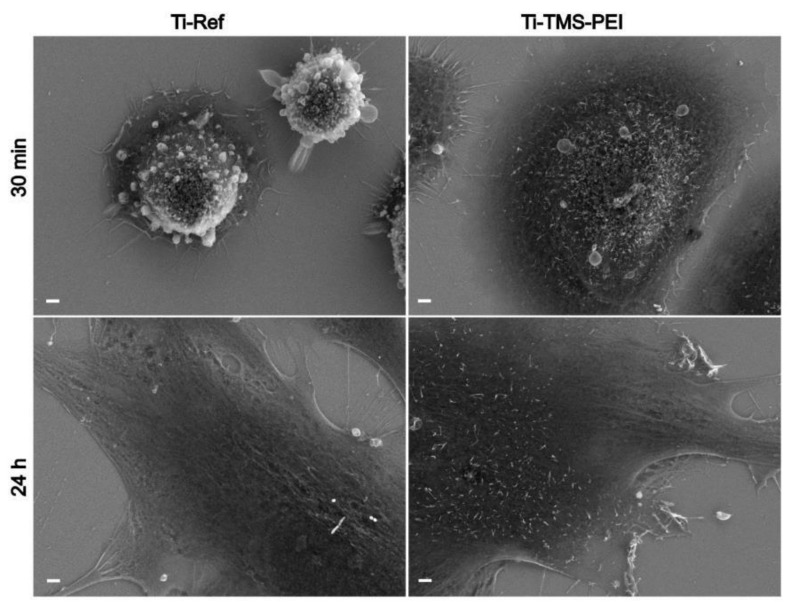
Morphology of MG-63 osteoblast-like cells on TMS-PEI-coated Ti for 30 min and 24 h versus the pure Ti-Ref. Note the considerably enhanced cell area after 30 min. No morphological differences are evident after 24 h (representative images of n = 3 independent experiments, scanning electron microscopy, FE-SEM Merlin VP compact, Carl Zeiss, scale bars 2 μm).

In addition, the outer surface morphology of MG-63s was compared using scanning electron microscopy (SEM) (Figure 3). It was evident that after 30 min of cultivation on Ti-Ref, the cells were still small in size, rounded, and spherical, while on the Ti-TMS-PEI coating, they were already growing flat and spread out. Also, the outer surface structure converged after 24 h between the different surfaces (Figure 3).

### 2.4. Migration Capacity of MG-63 Cells on Ti-TMS-PEI

**Figure 4:**
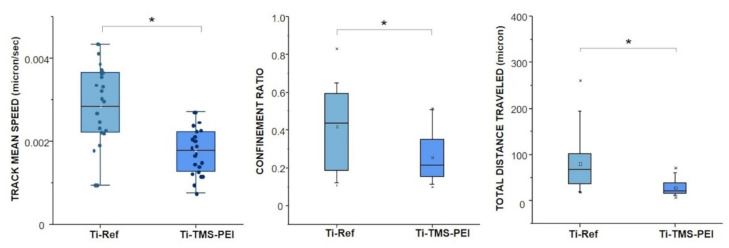
Speed and traveled distance of MG-63 cells adhering on pure Ti or TMS-PEI functionalized surfaces over 18 h. Cells were cultivated in an incubation chamber of an upright confocal laser scanning microscope LSM 800 (Carl Zeiss), and live cell imaging was performed. A decreased migration capacity of MG-63 cells on TMS-PEI compared to Ti-Ref could be observed after this longer cell contact on the amine-rich nanolayer (median ± IQR, x—outliner; non-parametric Mann-Whitney test, * *p* < 0.05).

To investigate the migration behavior of MG-63 cells on the TMS-PEI coating of Ti, live cell imaging of MG-63 cells on Ti-Ref and Ti-TMS-PEI surfaces was performed. The cells were cultured on the surfaces for 18 h, and the speed and distance of their movements were measured. The speed (0.003 µm/s for Ti-Ref, 0.0018 µm/s for TMS-PEI), the total distance traveled (65.6 µm for Ti-Ref, 22.1 µm for TMS-PEI), and the confinement ratio (0.45 for Ti-Ref, 0.21 for TMS-PEI) were significantly different. We observed a slower cell migration speed on TMS-PEI after the 18 h contact time (Figure 4).

**Figure 5:**
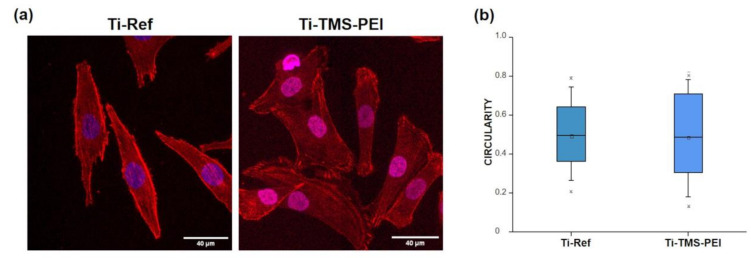
Circularity quantification over 18 h of cells adhering on pure Ti or TMS-PEI functionalized Ti surfaces. (**a**) Actin stained, living MG-63 cells on Ti-TMS-PEI and Ti-Ref for 18 h in an incubation chamber of an upright confocal microscope LSM 800 (Carl Zeiss; SPY650-FastAct™, red, for actin, SPY555-DNA™, blue, for nucleus, scale bars 40 µm). (**b**) Analysis of live cell imaging: no difference could be observed between the cell circularity after each cultivation condition (two-sample tests for variance at the level *p* < 0.05, n.s., median ± IQR, x—outliner).

In this context, the circularity of MG-63 cells was also determined after 18 h of cultivation on Ti-Ref and Ti-TMS-PEI (Figure 5). There were no differences in circularity between the two surface conditions (median: 0.48 ± 0.28 for Ti-Ref, 0.48 ± 0.39 for TMS-PEI) (Figure 5b). This result is in correlation with the spreading data after 24 h cultivation and underlines the findings that the positive influence of Ti-TMS-PEI coating on the spreading behavior of MG-63 cells is initially focused on the first cell–material contacts.

### 2.5. Bovine Serum Albumin (BSA) Adsorption and Desorption on Ti-TMS-PEI

The summary of the BSA concentrations on the desorption, adsorption, and washing protein solutions detected at UV 215 nm and the calculated amount of BSA adsorbed on the Ti-Ref and Ti-TMS-PEI surfaces are shown in Table 3. The Ti-TMS-PEI adsorbed a higher amount of BSA than Ti-Ref by a factor of 4.2.

**Figure 6:**
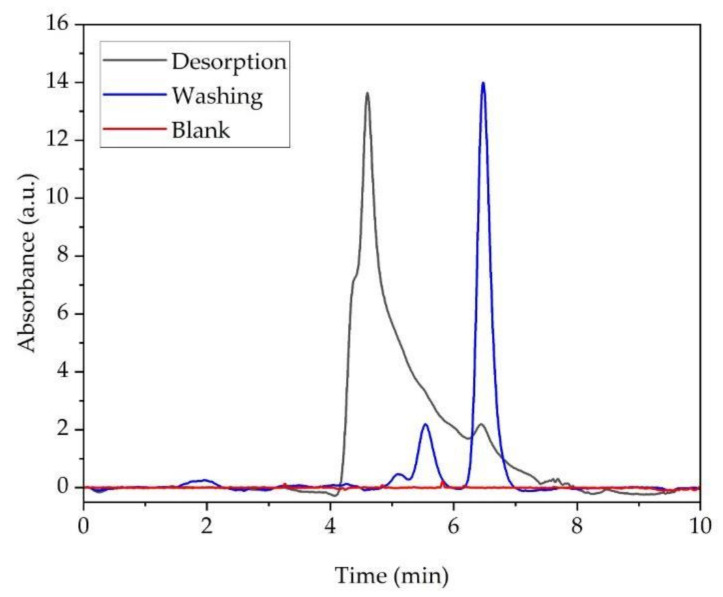
Representative chromatograms of a blank Ti-TMS-PEI subjected to the same solution used in the desorption process and the washing and the desorption solutions of BSA on Ti-TMS-PEI detected at UV 215 nm. The washing and desorption solutions were analyzed using size-exclusion HPLC with an Agilent AdvanceBIO SEC 300A 2.7 μm column as the stationary phase and 10 mM PBS + 0.1% SDS at a flow rate of 1.0 mL/min as the mobile phase. (a.u.: arbitrary unit).

Figure 6 shows the representative chromatograms of the washing and desorption solutions of Ti-TMS-PEI detected at UV 215 nm. The chromatogram of the adsorption solution is not shown here since it resembles the retention peaks of the washing solution but at much higher absorbance values, as evidenced by the BSA concentration values shown in Table 3. The observed peak retention times for the BSA oligomers, dimers, and monomers are 5.08 min, 5.53 min, and 6.48 min, respectively. The Ti-TMS-PEI induced aggregation of the BSA protein during the desorption process since the concentration of the BSA oligomers is greater than with the washing solution. The heating and the presence of 0.1% SDS in the solution allowed the denaturation of the protein desorbed from the surface. Protein aggregation occurred due to the high shear experienced by the substrate as it rubs against the bottom of the flask during agitation and also from the high concentration of adsorbed proteins due to the high density of amino groups in the Ti-TMS-PEI.

To verify that no protein aggregates and degradation products were released from a blank Ti-TMS-PEI, the blank sample that did not undergo the protein adsorption process was subjected to the same type of solution used in the desorption process. Figure 6 also shows that there were no proteins or degradation products released from the blank Ti-TMS-PEI, which supports the hypothesis that the observed protein aggregation was due to the interaction of the Ti-TMS-PEI with the BSA and the environment during the desorption process. The surface with high amino group density showed greater adsorption of the model protein used in this study, which correlates with the enhanced spreading of the MG-63s on the Ti-TMS-PEI.

### 2.6. Gene Expression of MG-63 Cells on Amine-Based Polymer Coating Ti-TMS-PEI

#### 2.6.1. Premature Activation of Erk1/2 Pathway after 4 h of MG-63 on Ti-TMS-PEI

**Figure 7:**
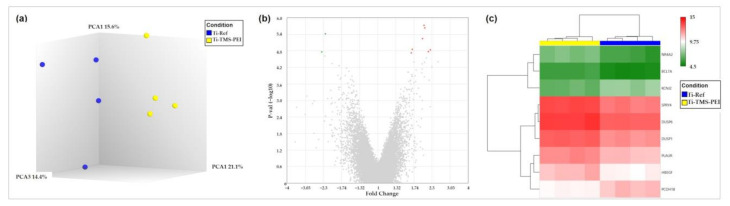
Gene expression of MG-63s after 4 h on amine-based Ti-TMS-PEI. Gene expression levels were analyzed via microarrays. (**a**) Principal component analysis shows distinct groups regarding global gene expression for Ti-Ref and Ti-TMS-PEI samples; (**b**) volcano plot with 7 overexpressed (red) and 2 downregulated (green) genes; (**c**) heat map shows the expression levels of the 9 differentially expressed genes in each sample of cells cultivated on Ti-TMS-PEI compared to Ti-Ref. Differential gene expression was defined by >1.5-fold difference with a *p*-value threshold of 0.05 and a false discovery rate (FDR) value threshold of 0.05. While two genes were downregulated (*PCDH18*, *KCNJ2*) after 4 h cultivation on Ti-TMS-PEI, seven genes were upregulated.

To gain deeper insight into the different cellular processes after surface coating with Ti-TMS-PEI, we performed microarray analyses after 4 h of cultivation with MG-63s. Significant differences appeared in the global gene expression of MG-63s compared to the uncoated Ti-Ref control (Figure 7a). Using stringent filtering criteria (>1.5-fold difference with a *p*-value cutoff of 0.05 and a false discovery rate (FDR) cutoff of 0.05), nine differentially expressed genes were identified (Figure 7, Table 4).

**Figure 8:**
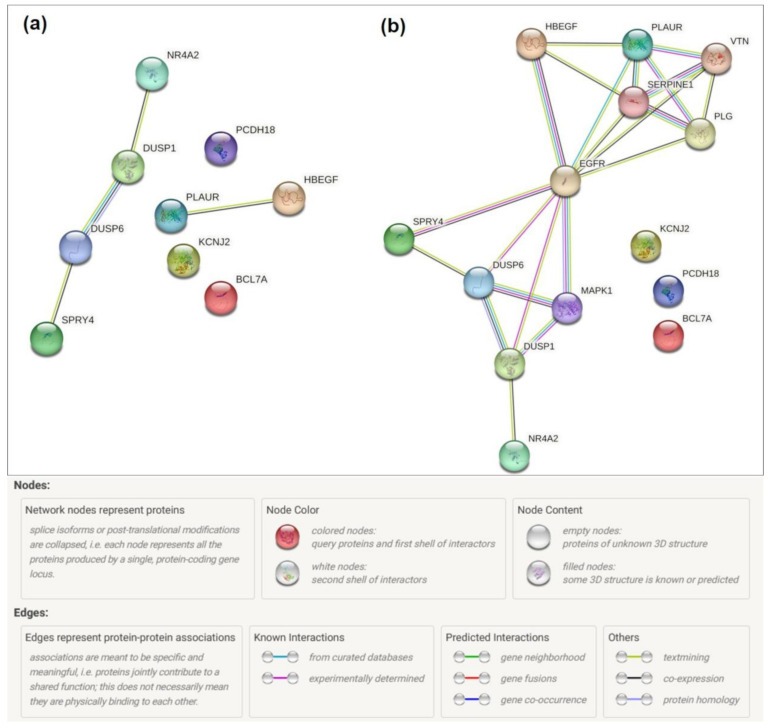
Network analysis of differentially expressed genes of MG-63 cells cultured on Ti-TMS-PEI vs. Ti-Ref for 4 h. (**a**) STRING analysis of differentially expressed genes showed only a few interactions. (**b**) STRING analysis of differentially expressed genes using intermediate components revealed an interaction network with epidermal growth factor receptor (EGFR) as the key component.

We performed a gene ontology (GO) annotation of each gene (Table 5) and gene set enrichment (Table 6). Also, we analyzed protein–protein interactions using the STRING database. Initially, very few interactions were detected (Figure 8a). Insertion of intermediate components revealed a network around the epidermal growth factor receptor (EGFR) (Figure 8b) containing all upregulated genes except *BCL7A*. A combination of all three analyses showed a starting down-regulation of the EGRF pathway, which includes cell proliferation.

#### 2.6.2. Premature Activation of Proliferation Pathways after 24 h Cultivation of MG-63s on Ti-TMS-PEI

**Figure 9:**
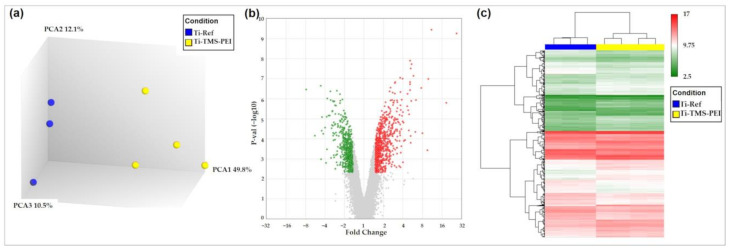
Gene expression of MG-63s after 24 h on Ti-TMS-PEI. Gene expression levels were analyzed via microarrays. (**a**) Principal component analysis shows distinct groups regarding global gene expression for Ti-Ref (n = 3) and Ti-TMS-PEI (n = 4) samples; (**b**) volcano plot with 732 overexpressed (red) and 566 downregulated genes (green); (**c**) heat map shows the expression levels of the 1298 differentially expressed genes in each sample of MG-63 cells on Ti-TMS-PEI compared to Ti-Ref. Differential gene expression was defined by >1.5-fold difference with a *p*-value threshold of 0.05 and an FDR value threshold of 0.05.

**Figure 10:**
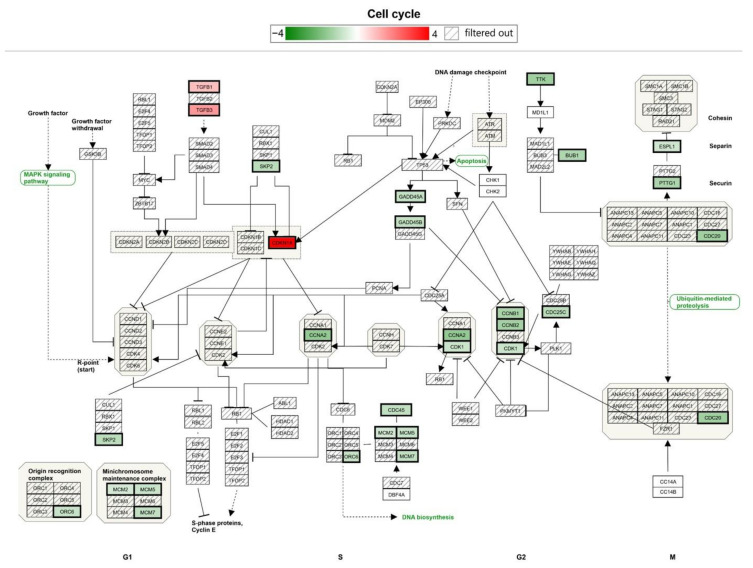
Expression of cell cycle associated genes after 24 h cultivation on Ti-TMS-PEI compared to Ti-Ref. Differential gene expression was defined by >1.5-fold difference with a *p*-value threshold of 0.05 and an FDR value threshold of 0.05. Three genes were upregulated (red boxes), and 18 genes were downregulated (green boxes) on Ti-TMS-PEI compared to Ti-Ref. While the upregulation is especially regarding the G1–S phase, the downregulation is mainly in the S–G2 phase of the cell.

MG-63 cells cultivated on Ti-TMS-PEI for 24 h showed 1298 differentially expressed genes compared to Ti-Ref (Figure 9). This represents a strong increase compared to the nine differentially expressed genes after 4 h cultivation on Ti-TMS-PEI. The number of overexpressed (732) and downregulated genes (566) is comparable. To evaluate which pathways are enriched in our subset of 1298 genes compared to all annotated genes on the chip, we did a WikiPathways analysis. This revealed, besides other findings, that the cell cycle signaling pathway was significantly enriched (Figure 10). Genes inducing mitosis and, therefore, the proliferation of MG-63s were downregulated, accompanied by those genes inducing cell cycle arrest, which were upregulated (Figure 10).

The pathway “osteoblast differentiation and related diseases” (Table 7) is also enriched. This means a larger amount of differentially enriched genes on Ti-TMS-PEI belong to this pathway (eight genes upregulated and five genes downregulated) than expected compared to Ti-Ref. Differentially expressed genes within this pathway demonstrate an upregulated differentiation capacity of MG-63s after 24 h on Ti-TMS-PEI.

## 3. Discussion

The initial step of osseointegration is the adhesion of osteoblasts [8]. To improve osseointegration of implants, it is necessary to promote initial cell adherence and thus accelerate the differentiation processes in bone tissue. During our screening of different amine-based nanocoatings, we identified Ti-TMS-PEI as a coating with moderately positive zeta potential, which is optimal for cell growth [14]. Only one other amine-based nanocoating showed a positive zeta potential, Ti-APTES-100, which can be classified as highly positive. However, Gruening et al. showed that surface charges above ζ +50 mV impaired the cell viability and proliferation after 24 h, although the initial cell spreading after short-time contact with those surfaces was increased [14]. Ti-TMS-PEI indicated a water contact angle in the optimal range [48] for cell adherence and growth regarding MG-63s. However, the Ti-GOPTS-bPEI and Ti-GOPTS-lPEI coatings also showed a WCA in this window around θ 50°. However, no positive zeta potential could be detected. In earlier studies, the cell behavior of MG-63s was shown to correlate with the material’s zeta potential but not with the water contact angle [14]. Cells preferred moderately positively charged surfaces. Here, the cell biological study including microarray analysis showed that Ti-TMS-PEI improved cell growth compared to Ti-Ref, e.g., after 4 h, the gene *HBEGF* was upregulated, which is involved in the positive regulation of cell migration, growth, and spreading. Interestingly, XPS data showed the highest amino group density of Ti-TMS-PEI (N/C value of 32%) compared to all other coatings, correlating with the positive effect on cell spreading.

Interestingly, MG-63s growing on amino-group-containing Ti-TMS-PEI showed differential expression of genes involved in cell growth and adherence already after 4 h cultivation. This indicates that the initial changes in cell adherence due to surface coating also occur at the level of gene expression. It remains open whether the altered gene expression causes the improved adherence or whether altered surface properties and, thus, enhanced cell–surface contacts cause signal transduction via the actin cytoskeleton into the nucleus [49]. There was likely an enhancement of proliferation and differentiation in MG-63 cells by mechanotransduction [50]. For example, MG-63s on Ti-TMS-PEI showed heparin-binding EGF-like growth factor (*HB-EGF*) overexpression, which promotes cell adherence, spreading, and cell migration [51].

Furthermore, the altered gene expression indicates a premature onset of proliferation of MG-63 cells on Ti-TMS-PEI. The cells have already completed the proliferation phase to initiate the differentiation phase. This could be due to a temporal advantage of the cells on the Ti-TMS-PEI surface, which is based on a faster adherence of the cells. In this process, the cells fall onto the surface by gravity and adhere at different rates within the first 15 min, depending on the surface characteristics. Cells that have just attached after 30 min thus show a rounded shape and low cell spreading. Amine-based coatings with positive charges, i.a., with plasma polymerized allylamine, can accelerate cell adherence and spreading [52], as also demonstrated with our amine-rich Ti-TMS-PEI coating here. However, the dependence of cell response to the amino group density was not evaluated in these earlier coating experiments. Also, in old age, the proliferation of osteoblasts is slowed down [53]. The effect of Ti-TMS-PEI shown here could thus compensate to some extent for the age-related slowing of osteoblast proliferation and thus promote osseointegration.

Gene expression data after 24 h cultivation of MG-63 cells showed overexpression of genes involved in differentiation. This indicates a temporally advanced differentiation of MG-63 cells on Ti-TMS-PEI relative to the uncoated Ti-Ref. Several of these genes mark the beginning of the differentiation phase. These include *HEY1* [40], which were also overexpressed in our study, and the Wnt/β-catenin signaling [36]. Differentiation markers like TGFβ/BMP, cytokine/JAK-STAT, and TNFα/RANKL signaling could not be identified, which makes sense since their activity increases at later stages of differentiation [54].

Furthermore, a suppressor of osteoblast differentiation is downregulated, namely fibroblast growth factor 5 (*FGF5*), supporting our findings of early initiation of differentiation. It is already known that cytoskeletal changes affect osteogenic differentiation, as occurs with improved spreading [49]. This we could also observe at the transcriptional level as an effect of TMS-PEI. The fact that differentiation on amine-based coatings is enhanced has already been shown [55], but only a few microarray data are currently available, especially regarding the amine-rich TMS-PEI coating.

Not only the cell spreading and gene expression were positively influenced by Ti-TMS-PEI, but also the protein adsorption. The Ti-TMS-PEI adsorbed a higher amount of BSA compared to uncoated Ti-Ref. Also, in comparison with other non-aminated materials, the amine-based surface had a higher adsorption performance; for example, it is at least 8 × higher than polydimethylsiloxane (PDMS) and 24 × higher than glass, where the amounts of BSA adsorbed on PDMS and glass, after 1 h of adsorption time, are 0.9 µg/cm^2^ and 0.3 µg/cm^2^, respectively [56]. The reason for higher BSA adsorption is the hydrophilic nature and the moderate positive surface charge of the amino groups. This result agrees with the work of Phan et al. on the investigation of BSA attachment onto self-assembled monolayers with different terminal functional groups such as –NH_2_, –COOH, –CH_3_, and –OH [21]. Protein aggregation occurred due to the high shear experienced by the substrate and the high concentration of adsorbed proteins due to the high density of amino groups in the Ti-TMS-PEI. Our results are also consistent with the study of Schvartz et al. [22] on the effect of agitation on protein aggregation and the work of Borzova et al. [23] on the thermal denaturation and aggregation of BSA.

Cell migration was also influenced by the amino-rich surface, as seen from the live-cell imaging results where we observed slower migration of MG-63 osteoblast cells on the amino-rich surface at the 18 h time point, which is consistent with the study of Faucheux et al. [28] on the dependence of fibroblast adhesion on the substrate’s chemistry. This suggests that amino-rich surfaces possess high cell adhesion capacity. The slow cell migration is also the effect of the moderately positive surface charges from the amine groups on the surfaces, and this is supported by the works of Gruening et al. [57], who showed that moderately positive surface charges exhibit a higher number of long actin filaments that promote cell–material adhesion. The changes in the cells’ circularity over time also correlate with the cell spreading and gene expression results.

It may be that for observations of the movement of whole cells or their intracellular components, the time point is decisive. We previously examined the mobility of the adaptor protein vinculin on a homogeneous plasma polymer nanolayer with amino groups and found that highly mobile vinculin contacts in the time frame 50–180 min after MG-63 cell settlement on this coating [58]. Positively charged surfaces as we have also introduced here with Ti-TMS-PEI seem to push the cells’ start, i.e., the initial phase of cell–material interaction which is also seen in our spreading data.

PEI itself has already been extensively researched, both as one of the first polymeric transfection reagents [59] and as a surface coating for implants [60]. In this context, PEI is a promising coating for improving surface properties and cell proliferation but shows a need for optimization in terms of reduced cytotoxicity and improved hemocompatibility. Therefore, it is reasonable to test to what extent the PEI modification in the form of TMS-PEI used in this work improves these properties. Our work has shown that the amine-rich TMS-PEI exhibits good biocompatibility over 24 h and significantly enhances the surface properties of titanium with respect to cell adherence and cell differentiation. For this purpose, long-term stability and cell compatibility tests should be carried out to test the extent to which the promising TMS-PEI coating can improve the osseointegration of titanium implants.

## 4. Conclusions

Amine-based nanocoatings with different amino group densities on Ti surfaces were generated. Physico-chemical characterization indicated for the majority of nanocoatings a hydrophilic state but only two with an additional positive surface charge. The moderately positively charged trimethoxysilylpropyl modified poly(ethyleneimine) (Ti-TMS-PEI) coating possessed the highest amino group density with an N/C of 32%. The cellular spreading on amine-based coatings correlated well with the amino group density. In addition, Ti-TMS-PEI improved cell spreading and surface anchorage. Differentially expressed genes within the pathway “osteoblast differentiation and related diseases” demonstrated an upregulated differentiation capacity of MG-63s after 24 h on Ti-TMS-PEI vs. uncoated Ti. The content of amino groups on biomaterial surfaces seems to be essential in improving cell contact at the interface and cell response.

## 5. Materials and Methods

### 5.1. Titanium Arrays

Planar silicon (Si) arrays sputtered with 100 nm titanium (Ti, purity > 99.999%) were obtained from the Center for Microtechnologies (ZfM, University of Technology Chemnitz, Chemnitz, Germany). The Si-Ti samples (Ti-Ref) were 675 ± 25 µm thick with a surface area of 10 × 10 mm or 10 × 20 mm. For the microgrooved samples (Ti-G20; groove and plateau width of 20 μm and step height of 2 μm), the Si wafers were microstructured using deep reactive-ion etching (DRIE; ZfM, Germany) [61] and finally sputter-coated with 100 nm Ti.

### 5.2. Coating with Collagen Type I (Col I)

For controls, Ti substrates were coated with the extracellular matrix protein collagen type I (Col I; 40 mg/cm^2^; rat tail, Corning, Discovery Labware, Bedford, MA, USA) and dried overnight at room temperature (RT), shaking at 300 rpm. The remaining acetic acid from the Col I solution was removed by rinsing twice with sterile phosphate buffer solution (PBS, Sigma-Aldrich, Darmstadt, Germany).

### 5.3. Amine-Based Nanocoatings

**Figure 11:**
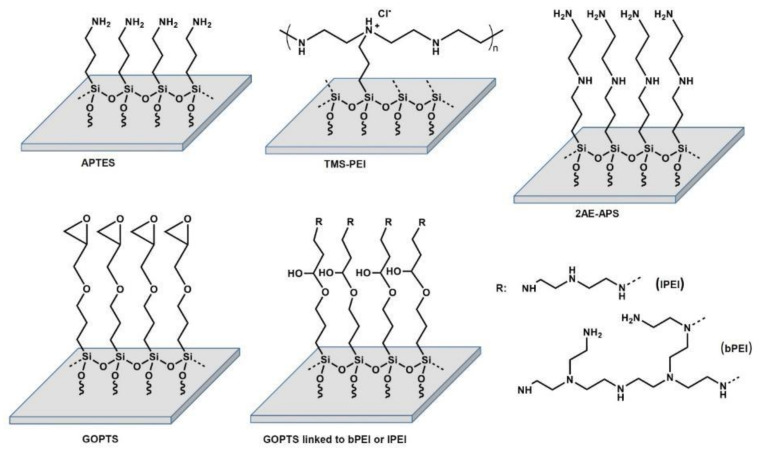
Amine-based nanocoatings on Si-Ti wafers. APTES: aminopropyltriethoxysilane TMS-PEI: trimethoxysilylpropyl modified poly(ethylenimine), 2AE-APS: n-(2-aminoethyl)-3-aminopropyltrimethoxysilane, GOPTS: (3-glycidyloxypropyl) trimethoxysilane, lPEI, bPEI: linear and branched (poly(ethyleneimine)).

Amine-based nanocoatings were prepared at INNOVENT e.V. (Jena, Germany; Figure 11).

#### 5.3.1. Cleaning and Activation Procedure

The Ti substrates were cleaned for 10 min in an isopropanol bath (Carl Roth, Karlsruhe, Germany) with sonication, dried with N_2_, and pre-activated by water vapor plasma treatment within a microwave oven at 1.5 mbar for 16 s.

#### 5.3.2. Trimethoxysilylpropyl Modified Poly(ethylenimine) (TMS-PEI)

The cleaned and pre-activated Ti substrates were modified with TMS-PEI/Isopropanol 50% (ABCR, Karlsruhe, Germany) after 1:1 dilution with isopropanol for 3 h at 60 °C. After carefully washing with isopropanol, the substrates were dried under a stream of pure nitrogen in a laminar flow box.

#### 5.3.3. Aminopropyltriethoxysilane (APTES)

Cleaned and pre-activated Ti surfaces were coated with 100 mM APTES (Sigma-Aldrich, Karlsruhe, Germany, Ti-APTES-100) in toluene (Alfa Aesar/Fisher Scientific, Kandel, Germany) for 3 h at 60 °C in an incubator at 35 rpm [2,14]. In another attempt, 1 mM APTES (Ti-APTES-1) in toluene was used as a coating mixture for 3 h at RT, according to Böhmler et al. [31]. Finally, after carefully washing with toluene, the coated substrates were dried under a stream of pure nitrogen in a laminar flow box (Herasafe KS12, Kendro, Langenselbold, Germany).

#### 5.3.4. (3-Glycidyloxypropyl)trimethoxysilane (GOPTS)

Cleaned and pre-activated Ti-substrates were treated with GOPTS (ABCR, Karlsruhe, Germany, pure) for 1.5 h at RT. After carefully washing with pure acetone (Carl Roth, Karlsruhe, Germany), the substrates were dried under a stream of pure nitrogen in a laminar flow box.

#### 5.3.5. Linear and Branched (Poly(ethyleneimine)) (lPEI, bPEI)

GOPTS-coated Ti-substrates, as prepared above, have been used for the immobilization of poly(ethyleneimines). A linear (Mn = 5 kDa, Merck, Darmstadt, Germany) and a branched (Mn = 10 kDa, Sigma-Aldrich, Steinheim, Germany) poly(ethyleneimine (PEI) was used. Immobilization of the GOPTS substrates were performed with 10 mg/mL PEI solution in a methanol/water mixture (9:1 *v*/*v*) for 1.5 h at RT. The excess polymer was removed by rinsing with a methanol/water mixture and methanol (Fisher Scientific, Schwerte, Germany) before the final substrates were dried under a stream of pure nitrogen in a laminar flow box.

#### 5.3.6. N-(2-Aminoethyl)-3-Aminopropyltrimethoxysilane (2AE-APS)

Cleaned and pre-activated Ti substrates were coated with 1 mM 2AE-APS (Sigma- Aldrich, Taufkirchen, Germany, Ti-2AE-APS-1) in toluene for 3 h at RT. After carefully washing with toluene, the substrates were dried under a stream of pure nitrogen in a laminar flow box.

### 5.4. Surface Characterization

#### 5.4.1. Wettability

The water contact angle (WCA) was determined by the sessile drop method using the Drop Shape Analyzer-DSA25 (Krüss, Hamburg, Germany) [62]. One ml drops of distilled water were deposited on the sample surface. Five drops were measured per sample (n = 3 independent experiments). Drop images were acquired with DSA25 digital camera. WCA values were calculated with the supplied software (ADVANCE, V.1.7.2.1, Krüss, Hamburg, Germany) using the optimal fit method (Ellipse) according to the curvature of the drop shape.

#### 5.4.2. Zeta Potential

As previously published, zeta potentials were measured with the SurPASS^TM^ system (AntonPaar, Ostfildern, Germany) and the associated software Attract 2.1 [62]. Streaming potentials were measured at pH 6.5–8.0 in a 1 mM KCl solution (VWR International, Darmstadt, Germany), and zeta potentials at pH 7.4 were calculated via a linear regression using the software GraphPad Prism version 6 (n = 3).

#### 5.4.3. X-ray Photoelectron Spectroscopy (XPS)

The samples were examined using an AXIS Ultra DLD XPS instrument from Kratos Analytical Ltd (Manchester, UK). The X-ray source emits monochromatic Al-Kα radiation (1486.8 eV). Overview and detail spectra were recorded with power and pass energy of 150 W and 160 eV and 225 W and 20 eV, respectively.

### 5.5. Cell Culture

MG-63 osteoblast-like cells (ATCC^®^ CRL-1427™, Manassas, VA, USA) were already extensively studied regarding their morphological and physiological stability [63]. Cells were cultured in Dulbecco’s Modified Eagle Medium (DMEM, 21063, Life Technologies, Renfrew, UK) with 10% fetal calf serum (FCS, Biochrom FCS Superior, Merck, Germany) and 1% gentamicin (Ratiopharm, Ulm, Germany) at 37 °C with 5% CO_2_.

### 5.6. Cell Area Determination

To analyze the extent of cell spreading, MG-63s were trypsinated and washed in a phosphate buffer solution (PBS, Sigma-Aldrich, Darmstadt, Germany). Then, membranes of living cells were stained with the PKH-26 General Cell Linker Kit (Sigma-Aldrich, Darmstadt, Germany) for 5 min at 37 °C in suspension. Cells were seeded onto the surfaces (50,000 cells/cm^2^), and cultivated for 30 min. Cells were washed twice with PBS, fixated with 4% paraformaldehyde (PFA, Merck, Darmstadt, Germany), embedded with a coverslip with Fluoroshield™ containing 4′,6-diamidino-2-phenylindole (DAPI, Merck, Darmstadt, Germany), and analyzed microscopically with the confocal laser scanning microscope LSM 780 (C-Apochromat 40×/1.20 water objective, ZEN black software 2011 SP4, all Carl Zeiss, Jena, Germany). The cell areas in μm^2^ of 50 cells per independent experiment were measured using ImageJ Version 1.46r.

### 5.7. Actin Cytoskeleton Staining

MG-63 cells were cultured for 30 min (50,000 cells/cm^2^) and 24 h (30,000 cells/cm^2^) on Ti, Ti-Col I, and Ti-TMS-PEI, respectively, and on Ti-G20, Ti-G20-Col I, and Ti-G20-TMS-PEI, respectively. Cells were then washed with PBS (Sigma-Aldrich, Germany), fixed with 4% PFA for 10 min at room temperature (RT), rewashed with PBS, and permeabilized with 0.1% Triton X-100 (Merck, Germany) for 10 min at RT. After another washing step with PBS, the cells were incubated with phalloidin-tetramethylrhodamine B isothiocyanate (phalloidin-TRITC, diluted 1:15 in PBS, Sigma-Aldrich) for 30 min at RT in the dark. Cells were again washed with PBS, embedded with DAPI-containing Fluoroshield™ and analyzed with the inverted confocal microscope LSM 780 (Carl Zeiss) using the ZEISS oil immersion objective (C-Apochromat 63) and the ZEN 2011 (black version) software (Carl Zeiss). To visualize cells’ actin cytoskeleton on Ti-G20 chips, confocal microscopy with three-dimensional (3D) z-stack generation was applied.

### 5.8. Scanning Electron Microscopy (SEM)

MG-63s were cultured on plain Ti chips coated with TMS-PEI for 30 min (50,000 cells/cm^2^) or 24 h (30,000 cells/cm^2^). Then, cells were washed two times with PBS and fixed with 2.5% glutardialdehyde (GDA, Merck, Germany) at 4 °C for 24 h. The samples were then dehydrated through a graded series of ethanol (30% for 5 min, 50% for 5 min, 75% for 10 min, 90% for 15 min, 100% 2× for 10 min) and dried in a critical point dryer (K 850, EMITECH, Berlin, Germany). Field-emission SEM (FE-SEM) observations were performed using the FESEM SUPRA 25 (Zeiss, Munich, Germany) with carbon coating at a low acceleration voltage (5 kV).

### 5.9. Gene Expression Profiling

For mRNA (messenger ribonucleic acid) expression profiles of MG-63 cells, human Clariom^TM^ S Arrays (Applied Biosystems, Thermo Fisher Scientific Inc., Santa Clara, CA, USA) were applied. For this, MG-63s (2 × 10^5^ cells) were cultured on Ti-TMS-PEI and the control Ti-Ref (quadruplicates from independent experiments) for 4 and 24 h, respectively. The Ti chips were transferred to fresh wells of a multiwell plate. This was done to prevent cells not adhering to the chip from being included in the analysis. Cells cultured on the surfaces were washed with PBS and lysed using 600 µL RLT buffer (Qiagen, Hilden, Germany) mixed with 1% β-mercaptoethanol. The following steps, i.e., extraction, quality control, labeling, and microarray processing, were carried out in the Core Genomic Facility at the Rostock University Medical Center. Sample preparation and microarray analysis were done as already published [64]. RNA purification included DNase I incubation on the spin column of the RNeasy Mini Kit (both from Qiagen). The total RNA samples were quantified spectrophotometrically (NanoDrop 1000, Thermo Fisher Scientific Inc., Waltham, MA, USA), and the integrity was controlled using the Agilent Bioanalyzer 2100 with the RNA Nano chip kit (both from Agilent Technologies, St. Clara, CA, USA). RIN (RNA integrity number) values between 9.6 and 10 were achieved. Each 200 ng whole RNA sample was used as starting material utilizing the GeneChip™ WT PLUS Reagent Kit (Applied Biosystems, Thermo Fisher Scientific Inc.). The microarray hybridization was done using Clariom S Array (Applied Biosystems, Thermo Fisher Scientific Inc.) according to the manufacturer’s instructions.

Generated CEL files were analyzed with the Transcriptome Analysis Console (TAC) 4.0 software (Applied Biosystems, Thermo Fisher Scientific Inc.). For probe set summarization, the Robust Multi-Chip Analysis, including the signal space transformation correction (SST-RMA), was used. This includes a background adjustment, a quantile normalization, and a log2 transformation of the data. Technical replicate groups were summarized by linear models for microarray data (LIMMA, eBayes) statistics. For one Ti-Ref sample, two out of four labeling controls on the array were out of the threshold. Therefore, this microarray sample was excluded from the analysis. For exploring the transcriptome changes induced by the TMS-PEI coating, differentially expressed genes were determined by comparing MG-63 on Ti-Ref versus on Ti-TMS-PEI. Differential gene expression was defined by >1.5-fold difference with a LIMMA *p*-value threshold of 0.05. Additionally, false discovery rates (FDR) were calculated to correct for multiple testing according to Benjamini and Hochberg [65], and an FDR q-value threshold of 0.05 was used according to recommendations in the literature [66]. The g:Profiler was applied for Gene set enrichment analysis [67], and the STRING database was used to analyze protein–protein interactions [68]. The raw and processed data can be found in the Gene Expression Omnibus (GEO) database under accession number GSE237945.

### 5.10. Live Cell Imaging

Ti samples were fixed in a 35 cm^2^ Petri dish with aquarium glue (AQUA-SILICONE, Den-Braven Sealants bv, Netherlands). Before use, surfaces were sterilized with 70% ethanol and washed with sterile PBS (Thermo Fisher Scientific, Lifetechnologies, France). One day before the live experiment, MG-63 cells were split into 6-well plates for further staining (Thermo Fisher Scientific). Four hours before the live experiment, one well of MG-63 cells from the 6-well plate was stained with 2 probes: SPY555-DNA™ to label the DNA (dilution 1:2000) and SPY650-FastAct™ (1:2000) to label actin (spirochrome, Switzerland) for 4 h. For the live experiment, the staining medium was kept and adjusted in concentration for 3.5 mL of medium. The cells were detached by trypsin, centrifuged, and resuspended in 200 µL of DMEM. Cells were observed using an upright confocal laser scanning microscope (CLSM) system LSM 800 (Carl Zeiss, Göttingen, Germany) and a 63× ON 1.0 plan-Apochromat water immersion objective lens (Carl Zeiss). Observations were performed over 18 h using an incubator chamber from Okolab^®^ where temperature and CO_2_ are controlled (37 °C, 5% CO_2_). Image acquisition was done with Zen 2.6 software using the experiment designer module. Parameters were adapted for each acquisition using fixed dimensions (4 tiles, 386 × 386 µm, zoom 0.5, pixel size 0.4 × 0.4 × 1 µm, z-stacks 70 slices) and laser excitation wavelength (555 nm and 639 nm). Each acquisition was analyzed with the TrackMate plugin in Fiji software (version 2.14.0) using the Trackmate-Cellpose and Trackmate-Stardist modules. Speed measurements, total distance traveled, and confinement ratio were quantified over 18 h for n = 20 cells within n = 2 independent experiments using Fiji software (version 2.14.0) and TrackMate-Stardist modules. The tracks with fewer than 5 spots were not considered. Cell circularity was quantified over 18 h for n = 20 cells within n = 2 experiments with the module Trackmate-Cellpose. Statistical analysis was performed with Origin software (9.9.0.220 Academic version).

### 5.11. Protein Adsorption

The protein adsorption and desorption performances of Ti-TMS-PEI were compared with the Ti-Ref. For the adsorption process, the samples were immersed in aqueous solutions (10 mM PBS) containing 200 μg/mL bovine serum albumin (BSA) at RT for 1 h. To desorb the adsorbed proteins from the surfaces, the samples were removed from the solution and washed with 10 mM PBS for 1 min to remove the loosely bound proteins. Then, the samples were dried with nitrogen gas before they were immersed in 10 mM PBS with 0.1% sodium dodecyl sulfate (SDS) at 60 °C for 1 h under ultrasonication to isolate the strongly bound proteins on the surface. The solutions from the adsorption, washing, and desorption were analyzed using size-exclusion high-performance liquid chromatography (HPLC) with an Agilent AdvanceBIO SEC 300A 2.7 μm column as the stationary phase and 10 mM PBS + 0.1% sodium dodecyl sulfate (SDS) at a flow rate of 1.0 mL/min as the mobile phase. The signals were detected by UV at 215 nm wavelength. The total adsorbed protein on the 1 cm^2^ sample was calculated by multiplying the BSA concentration from the desorption process (in µg/mL and determined via the HPLC) to the total amount of desorption solution used, which in this case is 3 mL.

### 5.12. Statistical Analyses

Statistical analyses were carried out with the software GraphPad Prism7 (GraphPad Software Inc., San Diego, CA, USA) or Origin (OriginLab, Northampton, MA, USA). The following data analyses were used (i) for cell spreading (Figure 1): 1-way ANOVA test, mean ± standard error of the mean (s.e.m.); (ii) for cell migration (Figure 4): non-parametric Mann-Whitney test, median ± interquartile range (IQR); (iii) for cell circularity (Figure 5): two-sample tests for variance, median ± IQR; (iv) LIMMA (linear models for microarray data) [69] and Benjamini and Hochberg [65] FDR for analyses of differential gene expression; (v) g:SCS (Set Counts and Sizes) correction method [70] for gene set enrichment analysis using g:Profiler [71]. *p*-values < 0.05 were considered to indicate significant differences.

## Figures and Tables

**Table 1:** Chemical characteristics of amino-group containing titanium coatings regarding water contact angle (WCA) and zeta potential (SurPass^TM^ system) (mean ± SD).

Surface Modification	WCA [θ in °]	Zeta Potential [ζ in mV]
**Ti-Ref** (titanium reference)	94.6° ± 3.5° 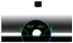	−81.2 ± 6.3 mV
**Ti-Col I** (collagen I)	53.1° ± 4.3° 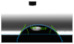	−2.8 ± 1.5 mV [14]
**Ti-2AE-APS** (N-(2-aminoethyl)-3-aminopropyltrimethoxysilane)	95.2° ± 0.4° 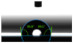	−11.5 ± 10.2 mV
**Ti-APTES-1** (1 mM aminopropyltriethoxysilane)	68.9° ± 6.3° 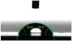	−20.6 ± 10.4 mV
**Ti-APTES-100** (100 mM aminopropyltriethoxysilane)	76.8° ± 1.7° 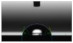	+51.4 ± 22.1 mV
**Ti-GOPTS-bPEI** (branched (poly(ethyleneimine)) on 3-glycidyloxypropyl)trimethoxysilane)	54.7° ± 2.0° 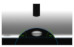	−34.2 ± 9.4 mV
**Ti-GOPTS-lPEI** (linear (poly(ethyleneimine)) on 3-glycidyloxypropyl)trimethoxysilane)	50.7° ± 3.8° 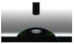	−48.9 ± 2.8 mV
**Ti-TMS-PEI** (trimethoxysilylpropyl modified poly(ethyleneimine))	53.6° ± 5.4° 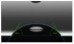	+11.7 ± 14.1 mV

**Table 2:** XPS results of amino-group-containing titanium coatings regarding common element composition (AXIS Ultra DLD). Note that further elements are not listed (Table S2). XPS results of coated Ti samples revealed a correlation between amino group density and cell spreading.

Coating	Ti [at-%]	N [at-%]	C [at-%]	N/C [%]		Spreading Fold Change of Col I	
Ti-APTES-1	14.13	3.40	40.56	8.38	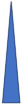	1.42	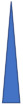
Ti-APTES-100	0.00	10.36	55.60	18.63		2.05	
Ti-2AE-APS-1	0.52	13.11	59.96	21.86		1.78	
Ti-GOPTS-l-PEI	22.21	4.14	18.16	22.80		1.37	
Ti-GOPTS-b-PEI	21.58	5.58	20.58	27.11		1.90	
Ti-TMS-PEI	21.39	7.38	23.04	32.03		2.29	

**Table 3:** Summary of the BSA concentrations on the desorption, adsorption, and washing protein solutions detected at UV 215 nm and the calculated amount of BSA adsorbed on the Ti-Ref and Ti-TMS-PEI surfaces (mean ± SD).

Substrates	BSA Concentration [µg/mL]			Adsorbed Amount
	Adsorption	Washing	Desorption	[µg/cm^2^]
Ti-Ref	189.1 ± 4.3	2.3 ± 0.1	0.6 ± 0.0	1.7 ± 0.0
Ti-TMS-PEI	181.8 ± 0.7	2.7 ± 0.0	2.4 ± 0.2	7.2 ± 0.7

**Table 4:** Differentially expressed genes of MG-63 cells after 4 h cultivation on Ti-TMS-PEI compared to Ti-Ref.

Gene	Fold Change *	*p*-Value	FDR *p*-Value	Description	Entrez ID
*SPRY4*	2.06	2.31 × 10^−6^	0.0247	sprouty RTK signaling antagonist 4	81848
*DUSP1*	2.03	1.87 × 10^−6^	0.0247	dual specificity phosphatase 1	1843
*PCDH18*	−2.23	3.77 × 10^−6^	0.0269	protocadherin 18	54510
*PLAUR*	1.99	5.60 × 10^−6^	0.0300	plasminogen activator, urokinase receptor	5329
*NR4A2*	2.17	1.66 × 10^−5^	0.0441	nuclear receptor subfamily 4, group A, member 2	4929
*HBEGF*	2.24	1.45 × 10^−5^	0.0441	heparin-binding EGF-like growth factor	1839
*BCL7A*	1.70	1.38 × 10^−5^	0.0441	B-cell CLL/lymphoma 7A	605
*DUSP6*	1.67	1.85 × 10^−5^	0.0441	dual specificity phosphatase 6	1848
*KCNJ2*	−2.36	1.71 × 10^−5^	0.0441	potassium channel, inwardly rectifying subfamily J, member 2	3759

* Upregulated genes in red, downregulated genes in green.

**Table 5:** Gene ontology annotation of differentially expressed genes of osteoblastic MG-63 cells cultivated on Ti-TMS-PEI for 4 h compared to Ti-Ref.

Gene *	Gene Ontology Annotation
*SPRY4*	negative regulation of ERK1 and ERK2 cascade; regulation of signal transduction; negative regulation of MAP kinase activity
*DUSP1*	inactivation of MAPK activity, negative regulation of ERK1 and ERK2 cascade; mitotic cell cycle arrest; regulation of mitotic cell cycle spindle assembly checkpoint; negative regulation of DNA biosynthetic process
*PCDH18*	Cadherin, homophilic cell adhesion via plasma membrane adhesion molecules; cell adhesion
*PLAUR*	plasminogen activator, urokinase receptor, CD59 antigen
*NR4A2*	nuclear receptor subfamily 4, group A, member 2
*HBEGF*	HBEGF: epidermal growth factor receptor signaling pathway; Fc-epsilon receptor signaling pathway; vascular endothelial growth factor receptor signaling pathway; neurotrophin TRK receptor signaling pathway; phosphatidylinositol-mediated signaling; MAPK cascade; activation of MAPKK activity; Ras protein signal transduction; insulin receptor signaling pathway; fibroblast growth factor receptor signaling pathway; positive regulation of cell migration; spreading of epidermal cells; cell chemotaxis; positive regulation of cell growth, positive regulation of cell proliferation
*BCL7A*	negative regulation of transcription
*DUSP6*	phosphatase activity; negative regulation in the MAPK pathway
*KCNJ2*	Kir2.1 inward-rectifier potassium channel; potassium ion transmembrane transport; magnesium ion transport; relaxation of cardiac muscle; relaxation of skeletal muscle; cellular response to mechanical stimulus

* Upregulated genes in red, downregulated genes in green.

**Table 6:** MG-63s on Ti-TMS-PEI for 4 h: Gene set enrichment analysis using g:Profiler revealed enrichment of gene ontology (GO) annotations regarding the inhibition of MAPK signaling within our subset of differentially expressed genes.

GO.ID	Description	*p*-Value	Genes
GO:0008330	protein tyrosine/threonine phosphatase activity	0.001	*DUSP1*, *DUSP6*
GO:0017017	MAP kinase tyrosine/serine/threonine phosphatase activity	0.002	*DUSP1*, *DUSP6*
GO:0033549	MAP kinase phosphatase activity	0.004	*DUSP1*, *DUSP6*
GO:0008138	protein tyrosine/serine/threonine phosphatase activity	0.028	*DUSP1*, *DUSP6*
GO:0051172	negative regulation of nitrogen compound metabolic process	0.008	*SPRY4*, *DUSP1*, *PLAUR*, *NR4A2*, *HBEGF*, *BCL7A*, *DUSP6*
GO:0031324	negative regulation of cellular metabolic process	0.011	*SPRY4*, *DUSP1*, *PLAUR*, *NR4A2*, *HBEGF*, *BCL7A*, *DUSP6*
GO:0051248	negative regulation of protein metabolic process	0.036	*SPRY4*, *DUSP1*, *PLAUR*, *HBEGF*, *DUSP6*
GO:1902532	negative regulation of intracellular signal transduction	0.045	*SPRY4*, *DUSP1*, *PLAUR*, *DUSP6*
GO:0001932	regulation of protein phosphorylation	0.047	*SPRY4*, *DUSP1*, *PLAUR*, *HBEGF*, *DUSP6*
GO:0043409	negative regulation of MAPK cascade	0.049	*SPRY4*, *DUSP1*, *DUSP6*
REAC:R-HSA-112409	RAF-independent MAPK1/3 activation	0.012	*DUSP1*, *DUSP6*
REAC:R-HSA-5675221	Negative regulation of MAPK pathway	0.045	*DUSP1*, *DUSP6*

**Table 7:** Differentially expressed genes regarding MG-63 osteoblast differentiation after 24 h cultivation on Ti-TMS-PEI.

Gene	Fold Change *	Description	Role in Osteoblast Differentiation	References	Entrez ID
*WNT5B*	−1.68	wingless-type MMTV integration site family, member 5B	WNT5B often functions as an antagonist of canonical WNT signaling; indication that WNT5B suppresses osteoblast differentiation	[35]	16265
*WNT2B*	−1.78	wingless-type MMTV integration site family, member 2B	increases during differentiation; promotes osteogenesis and regulates expression of Osterix and RUNX2, which drive differentiation	[36]	12781
*FGF2*	−2.18	fibroblast growth factor 2	enhances the osteogenic potential of BMSCs and their proliferative capacity. FGF2 induces bone and BMSC proliferation; proliferation factor for bone cells in cell culture; fibroblast growth factor 2 (FGF2) positively modulates osteoblast differentiation and bone formation	[37,38]	3676
*FGF5*	−1.60	fibroblast growth factor 5	promotes OS cell proliferation via MAPK signaling pathway; closely associated with poor differentiation	[39]	3683
*HEY1*	1.68	hes-related family bHLH transcription factor with YRPW motif 1	upregulated expression at the immediate early stage of BMP9-induced osteogenic differentiation; acts synergistically with Runx2 in BMP9-induced osteogenic differentiation	[40]	4880
*WNT9A*	3.23	wingless-type MMTV integration site family, member 9A	expression decreases during osteoblastic differentiation; Wnt9a knockout mice display bone malformations (low levels of bone ossification, indications of skeletal dysplasia); activates Loxl2 (collagen-cross-linking enzyme) which activates the biogenesis of connective tissue	[36,41,42]	12778
*WNT7B*	2.41	wingless-type MMTV integration site family, member 7B	enhancing factor for osteoblast differentiation, induces the non-canonical activation of mTORC1 and PKC δ signaling	[41]	12787
*WNT5A*	1.64	wingless-type MMTV integration site family, member 5A	involved in osteoblast differentiation; upregulates Lrp5/6; mediates the mechanical stretch-induced osteogenic differentiation of BMSCs	[43,44]	12784
*FGF7*	1.79	fibroblast growth factor 7	facilitates osteogenic differentiation of embryonic stem cells; activates ERK/Runx2	[45]	3685
*FGF18*	1.92	fibroblast growth factor 18	expressed in osteogenic mesenchymal cells and differentiating osteoblasts	[46]	3674
*PRKCH*	2.59	protein kinase C, eta	upstream activator of RUNX2 (essential transcription factor for osteoblast differentiation)	[47]	9403

* Upregulated genes in red, downregulated genes in green.

## Data Availability

The raw and processed data of the microarrays can be found in the Gene Expression Omnibus (GEO) database under accession number GSE237945. The other datasets presented in this study are available from the corresponding author on reasonable request. The dataset is stored on the local UMR server.

## Supplementary Materials

The following supporting information can be downloaded at: https://www.mdpi.com/article/10.3390/molecules28186505/s1, Table S1: Cell spreading data on polymer coatings; Table S2: Detailed XPS results of amino-group containing titanium coatings.
