# Supplementary material for: Response of Osteoblasts on Amine-Based Nanocoatings Correlates with the Amino Group Density
Source: Molecules. 2023 Sep 7;28(18):6505. doi: 10.3390/molecules28186505 (PMC10534789; doi:10.3390/molecules28186505)
Supplement: Supplementary file 1 [file molecules-28-06505-s001.zip › molecules-2556053_Table S1_Spreading data.pdf]

Table S1: Cell spreading data on polymer coatings.

**Ti-2AE-APS coating**				
**Cell area Ti-Ref [µm^2]**	**Cell area Ti-Col I [µm^2]**	**Cell area Ti-2AE-APS [µm^2]**	**Ti-2AE-APS/ Col I [fold change]**	**Ti-Ref/ Col I [fold change]**
434.1	635.8	1140.7	1.7	0.7
438.7	692.5	1302.8	1.8	0.6
499.6	681.6	1263.9	1.8	0.7
**1 mM APTES coating**				
**Cell area Ti-Ref [µm^2]**	**Cell area Ti-Col I [µm^2]**	**Cell area Ti-APTES-1 [µm^2]**	**Ti-APTES-1/ Col I [fold change]**	**Ti-Ref/ Col I [fold change]**
414.6	416.6	580.2	1.4	1
495.2	600.5	769.4	1.3	0.8
485.5	461.8	728.2	1.6	1.1
**100 mM APTES coating**				
**Cell area Ti-Ref [µm^2]**	**Cell area Ti-Col I [µm^2]**	**Cell area Ti-APTES-100 [µm^2]**	**Ti-APTES-100/ Col I [fold change]**	**Ti-Ref/ Col I [fold change]**
727.1	635.8	1151.0	1.8	1.1
602.0	602.4	1446.6	2.4	1
417.8	439.2	866.1	2	1
**Ti-GOPTS-b-PEI coating**				
**Cell area Ti-Ref [µm^2]**	**Cell area Ti-Col I [µm^2]**	**Cell area Ti-b-PEI [µm^2]**	**Ti-bPEI/ Col I [fold change]**	**Ti-Ref/ Col I [fold change]**
553.7	756.1	1290.6	1.7	0.7
481.7	702.0	1400.7	2	0.7
634.1	724.4	1402.4	2	0.9
**l-PEI coating**				
**Cell area Ti-Ref [µm^2]**	**Cell area Ti-Col I [µm^2]**	**Cell area Ti-l-PEI [µm^2]**	**Ti-lPEI/ Col I [fold change]**	**Ti-Ref/ Col I [fold change]**
553.7	756.1	963.4	1.3	0.7
481.7	702.0	858.6	1.2	0.7
634.1	724.4	687.4	1.6	0.9
**TMS-PEI coating**				
**Cell area Ti-Ref [µm^2]**	**Cell area Ti-Col I [µm^2]**	**Cell area Ti-TMS-PEI [µm^2]**	**Ti-TMS-PEI/ Col I [fold change]**	**Ti-Ref/ Col I [fold change]**
727.1	635.8	1632.2	2.6	1.1
602.0	602.4	1284.8	2.2	1
417.8	439.2	818,5	2.1	1
