# Supplementary material for: Response of Osteoblasts on Amine-Based Nanocoatings Correlates with the Amino Group Density
Source: Molecules. 2023 Sep 7;28(18):6505. doi: 10.3390/molecules28186505 (PMC10534789; doi:10.3390/molecules28186505)
Supplement: Supplementary file 1 [file molecules-28-06505-s001.zip › molecules_2556053_Table S2_XPS data.pdf]

Table S2: Detailed XPS results of amino-group containing titanium coatings regarding

common element composition (AXIS Ultra DLD).

	Ti [at-%]	C [at-%]	O [at-%]	Si [at-%]	N [at-%]	Rest [at-%]
**Ti-APTES-1** *(1 mM aminopropyltriethoxysilane)*	14.13	40.56	37.19	1.77	3.40	2.95
**Ti-APTES-100** *(100 mM aminopropyltriethoxysilane)*	0	55.6	20.58	13.46	10.36	0
**Ti-2AE-APS** (N-(2-aminoethyl)-3-aminopropyltrimethoxysilane)	0.52	59.96	16.73	8.77	13.11	0.91
**Ti-GOPTS-lPEI** (linear (poly-(ethyleneimine)) on 3-glycidyloxypropyl) trimethoxysilane)	22.21	18.16	51.05	1.09	4.14	3.35
**Ti-GOPTS-bPEI** (branched (poly(ethyleneimine)) on 3-glycidyloxypropyl)trimethoxy silane)	21.58	20.58	48.13	1.36	5.58	2.77
**Ti-TMS-PEI** (trimethoxysilylpropyl modified poly(ethyleneimine))	21.39	23.04	44.76	0.55	7.38	2.88
